# Identification of genes in HepG2 cells that respond to DNA methylation and histone deacetylation inhibitor treatment

**DOI:** 10.3892/etm.2014.1789

**Published:** 2014-06-18

**Authors:** QIANG SUN, YU XIE, GUOJING WANG, JIDONG LI

**Affiliations:** 1 General Surgery Department, The Second Artillery General Hospital of PLA, Beijing 100088, P.R. China; 2 Surgical Oncology Department, Chinese PLA General Hospital, Beijing 100853, P.R. China

**Keywords:** HepG2, epigenetics, DNA methylation, histone deacetylation

## Abstract

Previous studies have demonstrated that epigenetics has an important role in the regulation of gene expression in cancer. Epigenetics is the study of reversible, heritable changes in gene function, which occur independently from changes in the DNA sequence. DNA methylation and histone deacetylation are the two most important epigenetic modifications. DNA methylation was one of the first discovered epigenetic modifications and it may lead to changes in chromatin structure, DNA conformation and DNA stability, thereby controlling gene expression. Sample data on the HepG2 cell line from the Gene Expression Omnibus database under GSE5230 accession number were obtained and GEOquery and the limma package were then used to analyze the data and identify differentially expressed genes using Gene Otology. This was conducted in order to investigate the effect on gene expression of inhibiting DNA methylation and histone deacetylation, and to explore the potential role of epigenetics in the development and treatment of hepatic carcinoma. It was found that inhibition of DNA methylation and histone deacetylation affected not only substance metabolism, but also the immune activity in HepG2 cells. Furthermore, common target sites for transcription factors were identified in the differentially expressed genes. It may be concluded that the inhibition of DNA methylation and histone deacetylation contributes to the treatment of hepatic carcinoma and may provide a novel therapeutic strategy for the treatment of hepatic cancer.

## Introduction

Hepatic carcinoma is the sixth most common cancer worldwide and the third most common cause of mortality from cancer with 626,000 cases and 598,000 mortalities annually (1). In China, there are 360,000 cases of hepatic carcinoma and 350,000 associated mortalities a year (2), and hepatic carcinoma is the second most common cause of cancer-associated mortalities (1,3). Hepatitis B virus and aflatoxins are considered the major and common factors attributed to the etiology of liver cancer, and they can act individually or synergistically on the liver to cause cancer (4,5). Other factors, including hepatitis C virus, genetic susceptibility or genetic polymorphisms, may also have an important role in the etiology of liver cancer (6).

Previous studies have investigated the mechanism of hepato-carcinogenesis (7,8). The majority of these studies have focused on the genetic changes in key tumor suppressor genes and oncogenes; however, it has been suggested that epigenetic disruption of gene expression may also have an important role in the development of cancer (9). Epigenetic events have been found to be involved in the etiology of a wide variety of types of human cancer, including hepatic carcinoma. The current definition of epigenetics is the study of heritable changes in gene expression that occur independently from changes in the primary DNA sequence (10). The heritability of gene expression patterns is primarily mediated by epigenetic modifications, which include DNA methylation, chromatin remodeling, histone replacement and alterations to histone tails (8,11,12). DNA methylation is the most extensively studied epigenetic modification in mammals, and it provides a stable gene silencing mechanism that has an important role in the regulation of gene expression and chromatin architecture (10). Several studies have reported that there are somatically acquired DNA methylation changes in various tumor-suppressor genes and other cancer-associated genes (13,14). Histone deacetylation is a type of histone modification that may regulate key cellular processes, including transcription, DNA replication and DNA repair (15). DNA methylation and histone deacetylation may work independently or in concert to alter gene expression during tumorigenesis. Therefore, in the present study, the effect of inhibiting DNA methylation and histone deacetylation in HepG2 cells was investigated to determine the potential role of epigenetic modifications in the development and treatment of hepatic carcinoma, and to explore a novel therapeutic strategy for the treatment of hepatic carcinoma.

## Materials and methods

### Research materials and gene chip

In order to explore the effect of DNA methylation and histone deacetylation on hepatoma cells, the HepG2 cell line was used. The cells had been treated with 5-aza-2′-deoxycytidine (5-aza-dC; aza), trichostatin A (TSA), and a combination of aza and TSA to inhibit DNA methylation, histone deacetylation and both methylation and deacetylation, respectively. The gene expression profiles of the treated cells were compared with those of the control group to investigate the effects of methylation and deacetylation on liver cancer cells. GSE5230 sample data from the Gene Expression Omnibus (GEO) database was used (16), which included 4 gene chips of the treatment by aza, TSA, combination of aza and TSA and the control group, respectively.

### Acquisition of the differentially expressed genes

The samples were identified and the microarray data were analyzed using the R software (v.2.13.0) (17) platform, as well as GEOquery (18) and the limma package to further process the data. GEOquery obtains chip expression profiling data from the GEO database quickly, whilst limma can be used to statistically analyze the differentially expressed genes (19,20). The GEOquery package was used to obtain data of chip expression profiling that had already been preprocessed, and the chip data as transformed with log2. The expression profiles of the HepG2 cells treated with aza, TSA, aza and TSA and the control group were then compared, and the differentially expressed genes inhibited by methylation and acetylation were analyzed using the linear regression model package limma.

### Gene Otology (GO) analysis of the differentially expressed genes

In order to investigate the changes in the differentially expressed genes at the cellular level and their functional clustering, classification of gene function and position was performed using GO (21), using the GOEAST platform (22). In the present study, a hyper-geometric algorithm was selected for the statistical analysis. The entire microarray probe was used as a background control and the differentially expressed genes from biological processes were clustered; thus, the effect of these differentially expressed genes on the cells was determined.

### Biological pathway data

In order to investigate the changes induced in the cells as a result of the inhibition of DNA methylation and histone deacetylation at the molecular level, the effects of these modifications on biological pathways were examined. All metabolic and non-metabolic pathways were acquired from the current public open access database WikiPathways(http://www.wikipathways.org) (23,24), and the WikiPathways clustering analysis of differentially expressed genes was achieved through the Gene Set Analysis Toolkit V2 platform (25,26), in order to determine the changes in the signal pathways of HepG2 cells.

### Identification of potential target sites for regulatory transcription factors

Based on the gene annotation data arranged by the MSigDB (http://www.broadinstitute.org/gsea/msigdb/index.jsp) database, analyzed by gene abundance, with statistical calculations conducted with a hypergeometric algorithm and calibrated by Benjamini and Hochberg (BH) procedure, the potential target sites for regulation by transcription factors were obtained.

## Results

### Identification of differentially expressed genes following the inhibition of methylation and deacetylation

**Figure 1:**
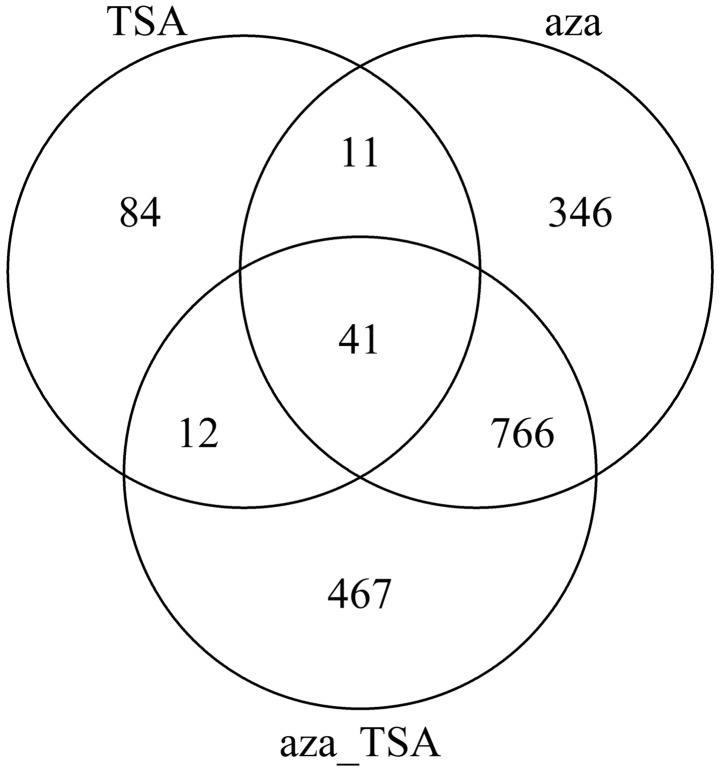
Number of differentially expressed genes following inhibition of epigenetic modifications. TSA, treatment with trichostatin A; aza, treatment with 5-aza-2′-deoxycytidine; aza_TSA, treatment with a combination of aza and TSA.

The data were analyzed using a t-test (20) modified by Bayesian model in order to obtain the differentially expressed genes. P-values were obtained for all the genes, and they were corrected as the false discovery rate (FDR). P<0.05 was considered to indicate a statistically significant difference. The numbers of genes with changes in expression levels are shown in Fig. 1.

As seen in Fig. 1, treatment with aza or TSA induced numerous changes in gene expression in HepG2 cells. The number of the altered genes following aza treatment was larger compared with that following TSA treatment. The results indicate that inhibition of DNA methylation and histone acetylation affects gene expression of HepG2 cells; however, methylation has a more significant contribution to the gene expression and regulation of liver cancer cells.

### Biological pathway enrichment regulated by DNA methylation and histone acetylation

Since the inhibition of DNA methylation and histone deacetylation caused changes in the expression in certain genes, changes in the biological pathways of hepatoma cells following the inhibition of DNA methylation and histone deacetylation was further investigated. The differentially expressed genes were selected and WikiPathways sub-pathway enrichment analysis was performed. The genes were clustered by hypergeometric algorithm and then multiplex detection was proofread using the BH algorithm in order to identify changes in the signaling pathways of hepatoma cells. Biological pathways significantly changed under the limiting conditions (corrected to P<0.1) with at least two genes in the signaling pathway are shown in Table I.

As the expression of only a few genes changed following treatment with TSA, clustering of only one signaling pathway, the transforming growth factor (TGF)-β signaling pathway, was observed. The TGF-β signaling pathway has very important roles in the body, including during embryonic development, cell growth, differentiation and apoptosis, as well as in intracellular metabolic balance. Therefore, histone deacetylation appears to have a critical effect on liver cancer cells.

DNA methylation was inhibited following treatment with aza, resulting in a series of genes being expressed differentially. Multiple biological pathways are associated with these differentially expressed genes, including signal transduction-related integrin-mediated cell adhesion, the adenosine monophosphate-activated protein kinase (AMPK) signaling pathway, the α6β4 signaling pathway, prostaglandin synthesis and regulation, the prolactin signaling pathway, metabolism-associated fatty acid β-oxidation, fluoropyrimidine activity, the drug-related irinotecan pathway and the cell motility-associated complement and coagulation cascades pathway.

The signaling pathways altered following treatment with Aza and TSA were broadly similar to those altered following treatment with aza alone, which include striated muscle contraction, the irinotecan pathway, AMPK signaling, the α6β4 signaling pathway, fluoropyrimidine activity and fatty acid β-oxidation. Furthermore, Aza and TSA co-treatment had a significant influence on fatty acid metabolism in HepG2 cells; however, mitochondrial long chain-fatty acid β-oxidation and fatty acid biosynthesis were indicated to be unaffected, with the exception of fatty acid β-oxidation.

### GO clustering of the differentially expressed genes

**Figure 2:**
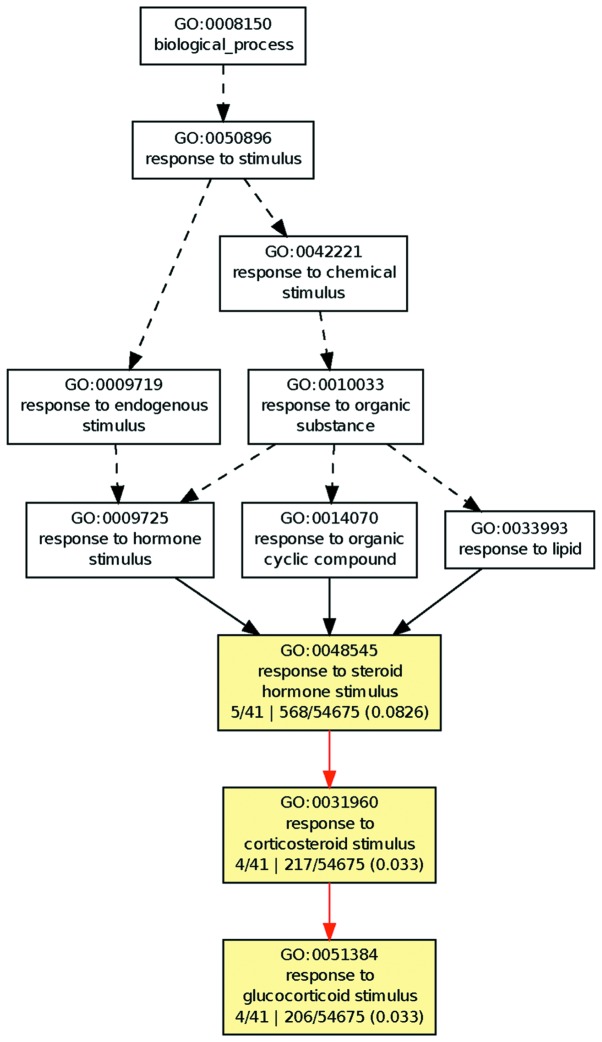
Clustering on physiological processes of the differentially expressed genes. The items with color were significant clustered (false discovery rate <0.05). The deeper the color, the stronger the significance.

In the organism, various means are required for regulation of the more important physiological processes. Therefore, the present study focused on the 41 genes expressed differentially for all three treatments. GO clustering was performed on their physiological processes using the GOEAST platform, as shown in Fig. 2.

The results demonstrated that these 41 genes clustered on the cell response to steroid hormones, in particular glucocorticoids. This suggests that DNA methylation and histone deacetylation have important roles in the regulation of the response to glucocorticoid in hepatoma cells.

### Analysis of target sites of the potential transcription factors

The spatial structure of the chromosome is altered as a result of the epigenetic modifications of DNA and histones, and this then alters the binding ability of trans-regulatory elements. As an important class of trans-regulatory elements, transcription factors may be a cause of the changes of gene expression following the inhibition of DNA methylation or histone deacetylation. Therefore, in the present study common binding sites for transcription factors shared by the differentially expressed genes were identified. Upstream sequences of the differentially expressed genes were used to investigate the potential target sites for the transcription factors, and the hypergeometric clustering algorithm was used, with proofreading of the P-value with the BH algorithm, and 10 target sites for the transcription factors were then identified (Table II).

## Discussion

The results from the WikiPathways clustering analysis demonstrated that inhibition of histone acetylation in HepG2 cells by exposure to TSA significantly affected the TGF-β signaling pathway, which indicates that histone deacetylation has an important role in the TGF-β signaling pathway in HepG2 cells. Furthermore, among the significantly upregulated genes in the TGF-β signaling pathway, lymphoid-enhancing factor 1 (LEF1) was of particular interest, as it is known to have a functional role in the Wnt/β-catenin pathway, another important pathway for tumor growth and invasion (27). Inhibition of histone acetylation in HepG2 cells may downregulate LEF1 expression, inhibiting the growth of HepG2 cells. In addition, bone morphogenetic protein 4 (BMP4) was found to be upregulated following inhibition of histone deacetylation. BMP4 is a member of the TGF-β family and is found in the liver. Previous studies have shown that BMP4 is constitutively expressed in the peribiliary stroma and endothelial cells in the liver and that its expression is downregulated following hepatectomy (28,29); in addition, BMP4 serves as an antiproliferative factor in hepatocyte proliferation (28).

Altering the TGF-β signaling pathway as a possible therapeutic treatment for cancer has been previously investigated in numerous studies (30,31). Therefore, the inhibition of histone acetylation in HepG2 cells to alter the TGF-β signaling pathway may provide targeted therapy for hepatic carcinoma; however, further investigation is required to determine the detailed mechanisms of TGF-β production and activation.

Treatment with aza and the combination of aza and TSA inhibited DNA methylation in HepG2 cells, which resulted in alterations in intracellular biological pathways, including integrin-mediated cell adhesion. Several of the signaling transduction pathways have important roles in growth, metabolism and regulation of differentiation in HepG2 cells. For example, TNFSF13, a member of the tumor necrosis factor family, was found to be upregulated following treatment with aza. TNFSF13 has a pathogenic role in the microenvironments of solid and hematological tumors (32). Elevated serum levels of TNFSF13 have been reported in oral cavity cancers (33), and are correlated with increased serum TNF levels, angiogenesis and poor prognosis in multiple myeloma (34). In addition, the inhibition of DNA methylation following treatment with aza and TSA resulted in changes in the AMPK signaling pathway. AMPK is a master regulator of energy homeostasis and is involved in the regulation of a number of physiological processes, including the β-oxidation of fatty acids, lipogenesis and protein and cholesterol synthesis. Previous studies have demonstrated that changes in these processes occur during cancer due to alterations in AMPK activity within cancer cells or in their periphery (35). The results of the present study demonstrated that tumor suppressor proteins tuberous sclerosis complex (TSC) 1 and 2, which are substrates of AMPK, were differentially expressed and tumor suppressor p53 was upregulated. In addition, DNA methylation has been investigated as a potential biomarker and therapeutic target in malignant tumors (36). Furthermore, inhibition of DNA methylation has a similar therapeutic effect as irinotecan, which is an effective drug for the treatment of certain types of cancer, including intestinal cancer and small cell carcinoma (37). Therefore, inhibition of DNA methylation and histone acetylation may provide a novel therapeutic treatment for hepatic carcinoma.

In addition, the clustering of the GO physiological processes of the 41 differentially expressed genes for the three treatment groups in the present study showed that the response of the HepG2 cells to glucocorticoids changed. Glucocorticoids are a class of steroid hormones secreted by zona fasciculata in the adrenal cortex, and have a role in the regulation of glucose and fat metabolism, and protein biosynthesis and metabolism (38,39). In addition, they may inhibit the immune response and have anti-inflammatory effects (38,40). Therefore, inhibition of DNA methylation and histone acetylation not only affects the metabolism of HepG2 cells, but also the immune activity.

Furthermore, a large number of the genes that were found to be differentially expressed following inhibition of DNA methylation and histone deacetylation may have the same target sites for transcription factors, and these sites may have an important role in the regulation of gene expression. For example, the differentially expressed gene BMP4 mentioned previously may be regulated by SMAD1. BMP4 signal transduction is dependent on SMAD phosphorylation via alk3 and SMAD signaling is associated with decreased hepatocyte proliferation following hepatectomy (41).

In conclusion, the present study identified a range of differentially expressed genes associated with DNA methylation and histone deacetylation blockage in HepG2 cells. Further studies of these genes and their regulation may aid in elucidating the underlying mechanism of the development of hepatocellular carcinoma.

## Figures and Tables

**Table I:** Changes in biological pathways following inhibition of DNA methylation and histone acetylation by aza and TSA.

Treatment	Pathway	P-value
Aza	Endochondral ossification	0.0816
	Integrin-mediated cell adhesion	0.0816
	Fatty acid β-oxidation	0.0816
	AMPK signaling	0.0816
	Fluoropyrimidine activity	0.0816
	Irinotecan pathway	0.0816
	α6β4 signaling pathway	0.0816
	Prostaglandin synthesis and regulation	0.0816
	Prolactin signaling pathway	0.0816
	Complement and coagulation cascades	0.0816
	Striated muscle contraction	0.0958
TSA	TGF-β signaling pathway	0.0015
	Striated muscle contraction	0.0504
	Irinotecan pathway	0.0504
	Mitochondrial LC-fatty acid β-oxidation	0.0588
TSA + aza	Fatty acid biosynthesis	0.0655
	AMPK signaling	0.0655
	α6β4 signaling pathway	0.0877
	Fluoropyrimidine activity	0.0877
	Fatty acid β-oxidation	0.0877

TSA, trichostatin A; aza, 5-aza-2′-deoxycytidine; AMPK, adenosine monophosphate-activated protein kinase; LC, long chain.

**Table II:** Potential target sites for transcription factors.

Drug	Target	P-value
Aza	hsa_RGAGGAARY_V$PU1_Q6	0.3087
	hsa_TATAAA_V$TATA_01	0.3087
	hsa_V$AP1_Q4	0.3544
	hsa_KTGGYRSGAA_UNKNOWN	0.4030
	hsa_V$E2F1DP1RB_01	0.4334
	hsa_CCCNNGGGAR_V$OLF1_01	0.4334
	hsa_V$E12_Q6	0.4334
	hsa_V$ER_Q6_02	0.4334
	hsa_V$AP1_C	0.4334
	hsa_V$CREBP1_01	0.4334
TSA	hsa_V$SMAD_Q6	0.0371
	hsa_V$IK1_01	0.0371
	hsa_V$MYCMAX_02	0.0371
	hsa_V$ZIC1_01	0.0371
	hsa_V$PBX1_01	0.0621
	hsa_V$FREAC3_01	0.0621
	hsa_V$USF_01	0.0621
	hsa_TGGAAA_V$NFAT_Q4_01	0.0621
	hsa_V$ARNT_01	0.0621
	hsa_V$GATA1_02	0.0621
Aza + TSA	hsa_TTTNNANAGCYR_UNKNOWN	0.0301
	hsa_CTGCAGY_UNKNOWN	0.7488
	hsa_KRCTCNNNNMANAGC_UNKNOWN	0.8112
	hsa_V$SRF_Q6	0.8487
	hsa_V$SRF_Q4	0.8487
	hsa_V$OCT1_03	0.8487
	hsa_V$ATF_01	0.8487
	hsa_V$HOXA4_Q2	0.8487
	hsa_V$TAL1BETAITF2_01	0.8487
	hsa_V$E2F_02	0.8487
